# Detection of Frost‐Damaged Maize Kernels for Food Quality Screening Using Hyperspectral Imaging and 2D‐COS‐Assisted Deep Learning

**DOI:** 10.1002/fsn3.72312

**Published:** 2026-09-18

**Authors:** Jiaxuan Nan, Dingguo Wang, Huihui Wang, Changxu Hu, Jiaxuan Li, Wuping Zhang, Fuzhong Li, Jiwan Han

**Affiliations:** ^1^ Software College Shanxi Agricultural University Taigu China; ^2^ Brunel London School North China University of Technology Beijing China

**Keywords:** deep learning, frost damage, hyperspectral imaging, maize kernels, two‐dimensional correlation spectroscopy

## Abstract

Maize is a crucial cereal crop susceptible to subzero temperature stress, which causes physicochemical damage to kernels and compromises their processing quality and food value. Traditional analytical methods are destructive, labor‐intensive, and unable to meet the requirements of rapid and high‐throughput quality screening in the food supply chain. This study established a nondestructive detection method combining hyperspectral imaging (900–1700 nm) and deep learning for the identification of frost‐damaged maize kernels. Multiple scattering correction (MSC) was verified as the optimal spectral preprocessing approach for improving spectral stability and classification performance. For feature extraction, Two‐Dimensional Correlation Spectroscopy (2D‐COS) effectively enhanced frost‐induced spectral variations by resolving overlapping and weak spectral responses, outperforming CARS and VCPA‐IRIV and extracting 432 characteristic bands associated with frost damage. The constructed 2D‐COS‐CNN model achieved a prediction accuracy of 0.9700 and a recall of 0.9647, with a calibration set accuracy of 0.9967, demonstrating excellent classification capability and reliable generalization performance. The integration of 2D‐COS‐based feature enhancement and CNN‐based nonlinear discrimination provides an effective strategy for rapid and nondestructive identification of frost‐damaged maize kernels, offering a reliable analytical tool for grain quality evaluation, damage‐level sorting, and processing suitability assessment in the food supply chain.

## Graphical abstract

This work presents a nondestructive strategy for identifying frost‐damaged maize kernels using hyperspectral imaging (900–1700 nm) combined with 2D‐COS‐assisted deep learning. MSC preprocessing was adopted, and 432 frost‐damage‐related characteristic bands were extracted by 2D‐COS. The 2D‐COS‐CNN model yielded high prediction accuracy (0.9700) and recall (0.9647). This approach enables rapid grain quality evaluation and damage‐level sorting in food supply chains.

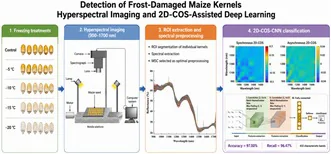

## 1 Introduction

As a major cereal crop worldwide, maize constitutes a cornerstone of global food supply, food processing, and the starch‐based ingredient industry (Hua et al. 2025). Nevertheless, the postharvest quality of maize kernels and their derived food products is extremely sensitive to subzero temperature stress. Subzero temperature incidents, including early autumn frost before harvest, frozen conditions during grain storage and transportation, invariably trigger irreversible physicochemical damage in maize kernels, including membrane lipid peroxidation, starch retrogradation, and protein denaturation, thereby severely degrading processing quality, nutritional value, and commercial grain quality (Zhang et al. 2022). These changes are particularly important for food processing applications because the structural integrity of starch granules, protein functionality, and lipid stability directly influence key processing characteristics, including gelatinization behavior, water‐holding capacity, and storage stability of maize‐derived products.

Under freezing circumstances, intracellular moisture in maize tissues crystallizes into ice crystals, which directly disrupt cell membrane integrity, accelerate the hydrolysis of starch into reducing sugars, promote protein denaturation and cross‐linking, and trigger lipid oxidation and free fatty acid accumulation. These biochemical alterations degrade pasting properties, reduce water‐holding capacity, impair fermentation performance, and may generate off‐flavors and discoloration in finished food products. Furthermore, frost‐damaged maize kernels are more susceptible to fungal colonization and mycotoxin contamination (such as aflatoxins and fumonisins) during subsequent storage, posing potential food safety risks (Zhang et al. 2025). Therefore, frost damage is not only an agricultural production problem but also a critical factor affecting food raw material quality, processing performance, and safety control throughout the food supply chain. Therefore, developing rapid, nondestructive methods for detecting frost‐induced quality deterioration in maize is of considerable importance to the food industry.

Conventional approaches for evaluating maize quality under temperature stress fall into two categories: physicochemical assays and sensory evaluations. Moisture content, crude protein, crude fat, and starch pasting properties are typically measured using standard reference methods (such as AACC and AOAC), which, while accurate, are time‐consuming, reagent‐intensive, and require well‐equipped laboratories (AACC International 2010). The falling number test and amylograph analysis, commonly used to assess starch damage in frost‐affected grains, demand specialized instrumentation and lengthy measurement cycles (Jia et al. 2016). Sensory evaluation by trained panels, although valuable for assessing overall acceptability, suffers from subjectivity and low throughput. In the grain trade, visual inspection remains the most widespread sorting practice, but it cannot reliably detect internal physicochemical damage invisible to the naked eye. Moreover, frost‐induced deterioration frequently occurs at the molecular and cellular levels before visible external changes become apparent, making conventional appearance‐based evaluation inadequate for early and accurate quality screening. These limitations create a well‐recognized gap between the need for rapid, high‐throughput quality screening in grain supply chains and the capabilities of existing analytical methods (Wang et al. 2020).

Hyperspectral imaging (HSI) has emerged as a powerful nondestructive analytical tool for food quality assessment, integrating spectroscopic information with spatial resolution to simultaneously capture the chemical fingerprints and morphological characteristics of food materials (Vincent and Dardenne 2021; Zhu et al. 2020). Operating in the near‐infrared range (900–1700 nm), HSI is sensitive to the overtones and combination bands of O–H, C–H, and N–H bonds, which are directly linked to the major macromolecular constituents of maize—starch, protein, and lipids. By capturing subtle spectral variations induced by freezing‐related biochemical changes (such as starch retrogradation, protein denaturation, and lipid oxidation), HSI offers the potential to discriminate frost‐damaged kernels from sound ones (Wongchaisuwat et al. 2025). Compared with traditional spectral techniques, HSI provides spatially resolved spectral fingerprints, enabling the characterization of heterogeneous physicochemical changes among individual kernels and offering significant advantages for rapid, noncontact inspection of food raw materials.

Temperature nonuniformity is a recognized challenge in low‐temperature food operations because products exposed to the same nominal process can experience different local thermal histories (Jia et al. 2026). In parallel, advances in broadband UV–Vis–NIR photodetection have improved sensitivity across wide optical windows (Li et al. 2023, 2024). Although those detector studies do not directly establish food‐quality performance, they illustrate the continuing expansion of optical sensing hardware relevant to rapid, noncontact inspection.

However, the high dimensionality and redundancy of hyperspectral data pose challenges for conventional multivariate analysis (Xu et al. 2023). The large number of highly correlated spectral variables may introduce redundant information and weaken damage‐related spectral responses, thereby reducing model robustness and limiting the interpretation of characteristic wavelengths. Therefore, effective spectral feature extraction strategies are essential for transforming complex hyperspectral information into highly discriminative representations associated with frost damage.

Deep learning (DL), particularly convolutional neural networks (CNN), provides a robust framework for automatic feature extraction and nonlinear pattern recognition from high‐dimensional spectral data, achieving superior performance in food quality classification tasks (Wen et al. 2026). Recent studies have successfully applied HSI combined with deep learning to detect fungal contamination in cereals, classify grain storage age, predict the nutritional composition of food commodities, and authenticate food products using spectroscopy‐based deep learning models (Ma et al. 2026), identify defective maize kernels (Xu et al. 2023), and discriminate corn kernel varieties using advanced deep learning architectures (Qi et al. 2025; Isik et al. 2024), demonstrating the broad applicability of this technical paradigm (Qi et al. 2026). Nevertheless, the classification performance of deep learning models is highly dependent on the quality of input spectral features. Combining effective spectral feature enhancement methods with deep learning algorithms may provide a more reliable approach for identifying subtle physicochemical variations caused by frost stress.

Two‐dimensional correlation spectroscopy (2D‐COS) is an effective spectral analysis technique that introduces external perturbations to transform conventional one‐dimensional spectra into two‐dimensional correlation maps. By enhancing spectral resolution and revealing asynchronous relationships among overlapping spectral responses, 2D‐COS can uncover weak and hidden spectral variations associated with biochemical changes. Therefore, integrating 2D‐COS with hyperspectral imaging may provide more informative characteristic features for deep learning‐based identification of frost‐induced quality deterioration.

The methodological novelty lies in using the ordered freezing‐temperature treatments as an explicit perturbation axis for 2D‐COS before CNN classification. Earlier NIR or HSI studies of frost‐damaged, nonviable, or defective maize kernels generally analyzed static one‐dimensional spectra or directly supplied spectra to chemometric or deep‐learning classifiers (Jia et al. 2016; Xu et al. 2023; Zhang et al. 2022). In contrast, the present workflow derives synchronous and asynchronous temperature‐response information, screens wavelengths from the resulting correlation structure, and then supplies those perturbation‐responsive variables to a 1D‐CNN. Thus, the contribution is the coupling of perturbation‐resolved spectral feature mining with nonlinear classification rather than the use of HSI or CNN alone.

Therefore, this study combines hyperspectral imaging technology with 2D‐COS‐assisted deep learning algorithms to classify maize kernels from five controlled freezing‐temperature treatments. To address the need for rapid quality screening of frost‐affected maize in food processing, this study was designed with the following objectives: (1) determine the optimal spectral preprocessing strategy and feature extraction method based on Partial Least Squares (PLS) prediction performance; (2) extract and visualize discriminative spectral features associated with frost damage and analyze feature representation using the t‐SNE algorithm; and (3) compare the performance of CNN with traditional machine learning models to evaluate their applicability for frost‐damage classification using hyperspectral datasets.

## 2 Materials and Methods

### 2.1 Sample Preparation

**FIGURE 1:**
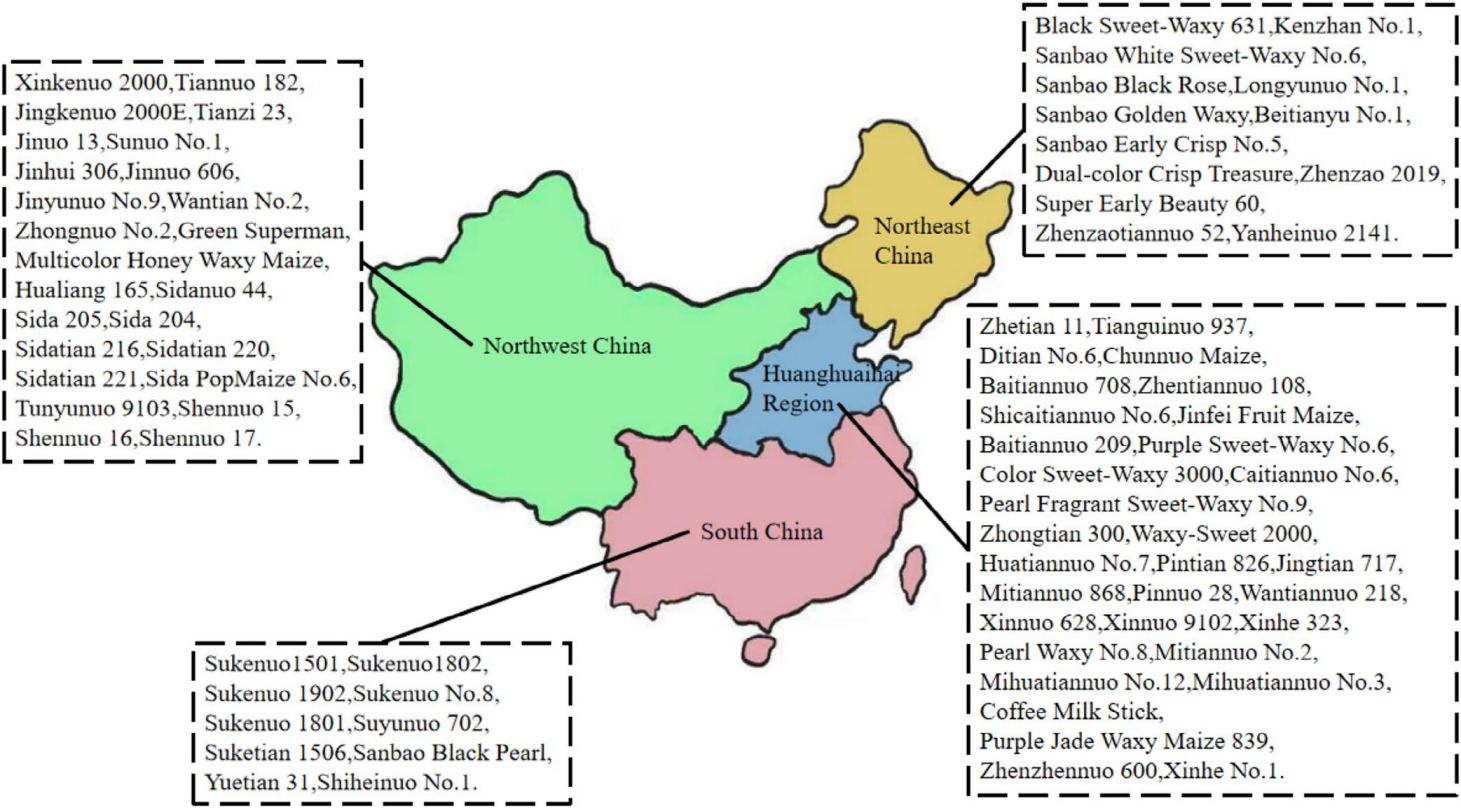
Distribution of maize cultivars across major growing regions in China.

Commercial maize kernels from 81 cultivars adapted to four major maize‐growing regions in China (South China, Huang‐Huai‐Hai River Basin, Northwest China, and Northeast China) were used in this study (Figure 1).

These cultivars were selected to represent the diversity of maize germplasm and regional environmental adaptation characteristics in China, thereby improving the representativeness of the experimental samples. For each of the 81 cultivars, five kernels were selected and one kernel was randomly allocated to each of the five treatment groups, yielding 81 kernels per group and 405 kernels in total. One group was maintained under normal conditions as the untreated control. The other four groups were subjected to controlled freezing treatments at −5°C, −10°C, −15°C, and −20°C for 10 h, respectively, to simulate different levels of low‐temperature stress during the postharvest period.

After cold treatment, all kernels were transferred to dry and ventilated conditions at 25°C and 50% relative humidity for 1 week. This recovery period was implemented to eliminate transient temperature effects and minimize spectral variations caused by rapid changes in surface moisture, allowing the hyperspectral signals to more accurately reflect the internal physicochemical alterations induced by frost damage. The incubation stabilized kernel ambient temperature and reduced experimental errors caused by moisture reabsorption.

Before freezing, initial moisture content, physiological maturity, kernel dimensions, and pre‐experimental storage history were not measured or standardized as independent covariates because the materials were commercial kernels selected to maximize cultivar diversity. All groups nevertheless followed the same allocation, freezing duration, recovery environment, and imaging protocol. Residual variation in these unmeasured initial properties may contribute to spectral differences and is a limitation of the present study.

The five class labels used in this study denote the imposed temperature treatments (untreated control, −5°C, −10°C, −15°C, and −20°C for 10 h) rather than independently quantified biological frost‐damage levels. Accordingly, classification performance should be interpreted as discrimination among controlled freezing‐temperature treatments. No parallel reference measurements of moisture distribution, starch gelatinization or pasting behavior, protein functionality, lipid oxidation, or membrane injury were collected; therefore, the spectral assignments to these mechanisms remain literature‐supported interpretations rather than direct causal validation.

### 2.2 Hyperspectral Image Acquisition and Correction

**FIGURE 2:**
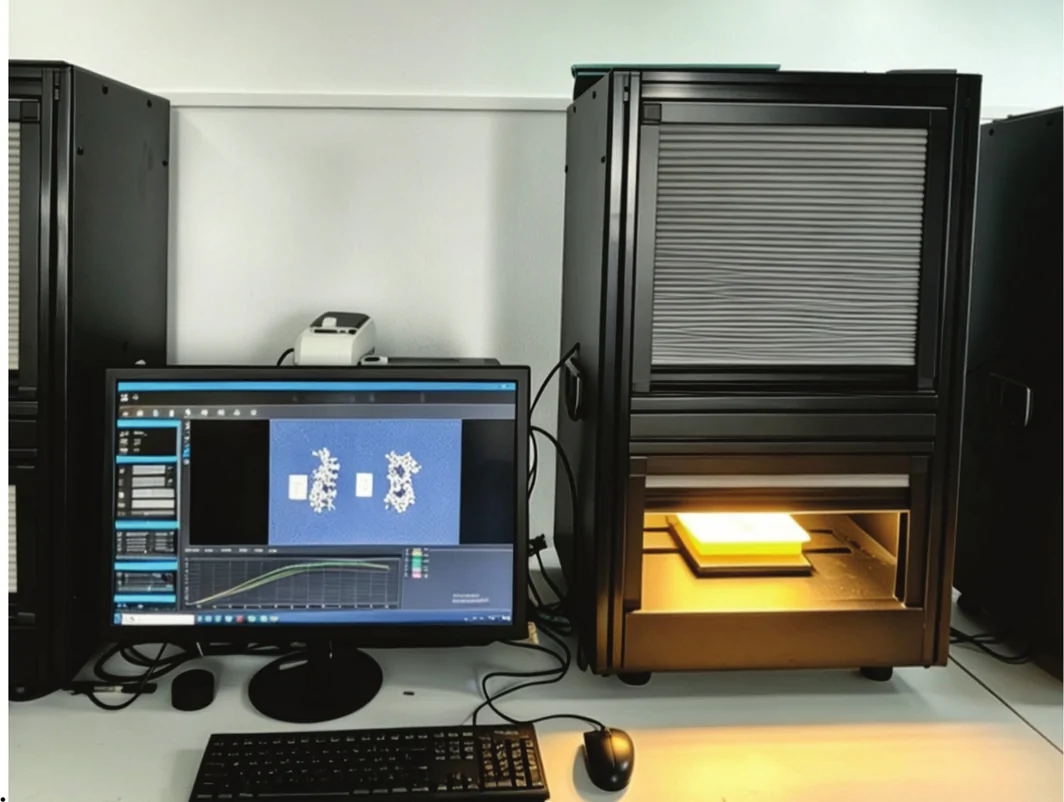
Hyperspectral data acquisition system.

The hyperspectral imaging system consisted of a uniform illumination source, a spectral camera, a computer, and corresponding control software (Figure 2). The camera model was EX‐9170, covering a spectral range of 900–1700 nm with a spectral sampling interval of 0.8 nm, a spectral resolution of ≤ 6 nm, a frame rate of 20 fps, a spatial resolution of 1280 pixels, and 1024 spectral channels. The system was equipped with a 600 W air‐cooled halogen lamp, a 1‐in. C‐mount 12 mm F1.4 lens, and a 20 × 20 cm motorized translation stage. All components were installed inside an optical dark box (47.5 × 55.5 × 87.5 cm) to minimize interference from external light sources. Hyperspectral images were acquired using a push‐broom scanning mode, in which the translation stage moved continuously beneath the camera to obtain spatially resolved spectral information of individual maize kernels.

Before image acquisition, the hyperspectral imaging system was preheated for 30 min to eliminate baseline drift and reduce systematic noise caused by unstable illumination and detector response. Subsequently, camera focusing and system parameter adjustments were performed to ensure consistent image quality throughout the experiment. All samples were placed on a low‐reflectance black background to enhance the contrast between maize kernels and the background, facilitating subsequent image segmentation and spectral extraction.

The original hyperspectral images were corrected to minimize the influence of dark current noise, illumination fluctuations, and instrument‐related errors. Following standard hyperspectral imaging calibration procedures, radiometric correction, spectral calibration, and geometric correction were considered during system operation, while reflectance calibration based on white and dark references was applied as the primary correction method in this study. This procedure ensured that the obtained spectra represented the intrinsic optical characteristics associated with frost‐induced biochemical changes rather than instrumental variation.

Under identical acquisition conditions, the image collected from a standard white reference board was defined as $R_{w}$, while the image obtained with the light source turned off and the camera lens covered was defined as $R_{d}$. The corrected reflectance spectrum was calculated according to Equation (1):

(1) $$\;R=\frac{R_{r}-R_{d}}{R_{w}-R_{d}}$$

where R is the spectral reflectance corresponding to each pixel, $R_{r}$ is the initial spectral data, $R_{d}$ is the dark reflectance images, $R_{w}$ is the white reflectance images.

### 2.3 Data Analysis

#### 2.3.1 Spectral Data Extraction and Preprocessing

The corrected hyperspectral cube data were obtained after reflectance calibration. The dataset consisted of 8000 rows, 1280 columns, and 1024 spectral bands, containing both spatial and spectral information of maize kernel samples. All image processing, spectral extraction, and data analysis procedures were performed using MATLAB R2023a.

**FIGURE 3:**
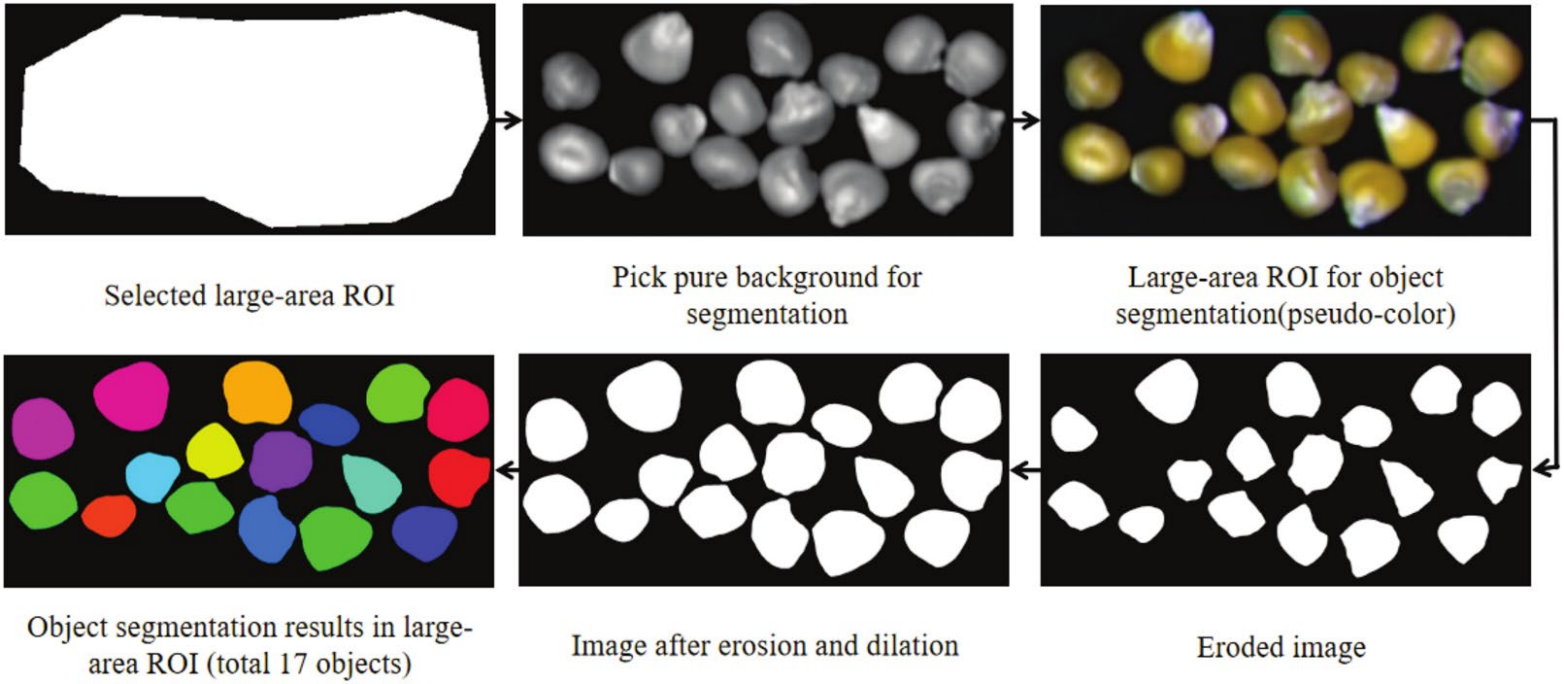
Data collection and processing process.

As illustrated in Figure 3, the spectral extraction process consisted of four sequential steps. First, the three‐dimensional hyperspectral data cube was imported, and pseudo‐color images were generated based on the full‐band average reflectance image. This visualization step preserved the overall spatial distribution information of samples and provided an intuitive basis for subsequent segmentation without affecting the original spectral information. A representative group of maize kernels from one cultivar containing five temperature treatments was selected as an example to demonstrate the complete processing workflow.

A large region of interest (ROI) containing all maize kernels was initially defined manually through human–computer interaction to exclude irrelevant regions and reduce background interference. Subsequently, a representative pure background area distant from the kernels was manually selected to characterize the spectral distribution of the background. Because the maize kernels exhibited distinct spectral responses compared with the low‐reflectance background, an adaptive threshold was calculated based on the background pixel distribution to improve the accuracy of kernel‐background separation. The large ROI and pure‐background region were selected once for each image by an operator using the same visual criterion: the ROI enclosed all kernels without contact with the image boundary, and the background patch contained no kernel pixels or visible illumination artifacts.

Within the selected ROI, the maximum between‐class variance method (Otsu thresholding) was applied to automatically determine the optimal segmentation threshold. Binary segmentation was then performed, followed by morphological operations including erosion and dilation to remove isolated noise pixels and refine the kernel boundaries. The morphological parameters were carefully adjusted to preserve the complete morphology of individual kernels while minimizing the loss of edge information. Otsu thresholding was calculated from the gray‐level distribution within each selected ROI rather than from a fixed global intensity value. The subsequent erosion‐dilation settings were adjusted empirically during the original workflow, but the exact structuring‐element size and iteration count were not archived. Consequently, operator dependence and possible loss of boundary pixels cannot be excluded; future implementations should record these parameters and compare the masks against manually annotated kernel boundaries. Edge‐aware or multiscale segmentation methods may further reduce the loss of small or ambiguous objects (Al‐Ghaili et al. 2020; He et al. 2023).

Connected component analysis was subsequently performed to automatically identify and label individual maize kernels. The complete pixel area corresponding to each kernel was regarded as an independent ROI. Finally, the spectral information of all pixels within each kernel ROI was extracted, and the average reflectance spectrum was calculated as the characteristic spectral profile of each sample. Averaging pixel‐level spectra within each kernel ROI reduced random pixel fluctuations caused by surface heterogeneity and improved the robustness of subsequent classification models. The extracted wavelength information and spectral datasets were integrated and exported into standardized files for further preprocessing and modeling.

Spectral preprocessing is an essential step in hyperspectral analysis because raw spectral signals often contain noise, scattering effects, and baseline variations caused by differences in sample morphology, surface structure, and illumination conditions. In this study, three commonly used preprocessing methods were investigated, including Savitzky–Golay (SG) smoothing, Multiple scattering correction (MSC), and first derivative transformation.

Savitzky–Golay (SG) smoothing reduces high‐frequency random noise while maintaining the original spectral profile and absorption characteristics through local polynomial fitting within a moving window (Savitzky and Golay 1964). MSC corrects multiplicative and additive scattering effects by transforming individual spectra according to the average reference spectrum, thereby improving the comparability of spectral responses among samples with different physical structures (Li et al. 2021). This characteristic is particularly important for maize kernels because frost damage may alter surface morphology and internal tissue structure, resulting in additional scattering variations unrelated to chemical composition.

The first derivative method eliminates baseline drift and enhances subtle spectral variations by converting gradual spectral changes into differential signals, allowing weak absorption characteristics associated with biochemical changes to be more effectively identified (Li et al. 2021). These preprocessing methods were compared based on subsequent classification performance to determine the optimal spectral enhancement strategy for frost‐damaged maize kernel identification.

#### 2.3.2 Characteristic Wavelength Extraction

To reduce spectral redundancy and improve model efficiency, three characteristic wavelength selection approaches were compared in this study, including Competitive Adaptive Reweighted Sampling (CARS), Variable Combination Population Analysis–Iteratively Retaining Informative Variables (VCPA‐IRIV), and Two‐Dimensional Correlation Spectroscopy (2D‐COS).

CARS is an effective variable selection algorithm based on the principle that the importance of variables is positively correlated with the absolute values of regression coefficients. It integrates Monte Carlo sampling, adaptive reweighting, and Partial Least Squares (PLS) regression to establish an iterative optimization framework. During each iteration, variables with higher contribution coefficients are preferentially retained, whereas variables containing redundant or irrelevant information are gradually eliminated. Therefore, CARS can efficiently identify informative wavelengths strongly associated with sample classification while maintaining the essential spectral characteristics of the original dataset (Song et al. 2024).

The VCPA‐IRIV method combines Variable Combination Population Analysis (VCPA) and Iteratively Retaining Informative Variables (IRIV) to achieve a two‐stage wavelength optimization strategy. In the first stage, VCPA performs preliminary variable compression through population‐based sampling and statistical evaluation, reducing the dimensionality of hyperspectral variables. Subsequently, IRIV further identifies informative wavelengths through iterative elimination of non‐contributing variables. This combination of coarse screening and fine optimization enables VCPA‐IRIV to achieve a balance between information preservation and dimensionality reduction in high‐dimensional spectral datasets (Tu et al. 2025).

In this study, two‐dimensional correlation spectroscopy (2D‐COS) was introduced as a spectral feature mining strategy to identify wavelength variables associated with frost‐induced biochemical variations. Unlike conventional wavelength selection methods that mainly rely on statistical correlations within static spectra, 2D‐COS introduces external perturbations and evaluates the dynamic spectral responses of samples, thereby enhancing the resolution of weak and overlapping spectral features. Temperature was selected as the external perturbation factor because different freezing treatments induced progressive physicochemical changes in maize kernels.

By applying a perturbation‐dependent transformation, 2D‐COS converts conventional one‐dimensional spectra into two‐dimensional synchronous and asynchronous correlation spectra with two independent wavelength axes. The mathematical expression of 2D‐COS is defined as follows:

(2) $$\Phi \left(\nu_{1},\nu_{2}\right)+\mathrm{i\Psi}\left(\nu_{1},\nu_{2}\right)=\frac{1}{\mathrm{\pi T}}\int_{0}^{\infty }\tilde{Y}\left(\nu_{1},\omega \right)\cdot {\tilde{Y}}^{*}\left(\nu_{2},\omega \right)d\omega$$

where represents the synchronous correlation intensity, represents the asynchronous correlation intensity, is the Fourier transform of the dynamic spectrum at wavenumber, is the complex conjugate of the Fourier transform, and is the Fourier frequency. This enables the effective resolution of severely overlapped or weakly distinguishable weak signal peaks embedded in raw spectra (Aït‐Kaddour et al. 2024).

The synchronous spectrum reflects the overall sensitivity of spectral variables to temperature‐induced perturbations through auto‐peaks, while the asynchronous spectrum provides additional information regarding the sequential response behavior of different molecular groups. Therefore, the combination of synchronous and asynchronous information provides a more comprehensive description of frost‐induced molecular changes, including variations in water status, carbohydrate structure, and protein‐related chemical bonds.

To quantitatively screen informative wavelengths, a comprehensive scoring strategy was established by integrating normalized synchronous intensity and asynchronous correlation significance. Wavelength variables with a comprehensive score greater than 0.5 were selected as characteristic bands. This strategy retained spectral variables with high sensitivity to freezing stress while reducing the influence of noise and redundant information, thereby providing more discriminative input features for subsequent classification models. The cutoff of 0.5 was defined as the midpoint of the unit‐normalized combined score and was fixed before classifier evaluation; prediction‐set labels were not used to tune this cutoff. The 432 wavelengths were selected once from the calibration data under this fixed rule. Selection stability across repeated resampling was not evaluated, and no additional exclusion window was imposed near the detector limits around 900 and 1700 nm. Because the raw spectra show increased edge noise in these regions, any retained edge wavelengths should be interpreted cautiously and formally assessed in future stability‐selection analyses.

### 2.4 Discriminant Model

To evaluate the feasibility of hyperspectral imaging combined with spectral feature extraction methods for frost‐damaged maize kernel identification, three classification models were constructed, including two traditional machine learning algorithms and one deep learning algorithm: Linear Discriminant Analysis (LDA), Support Vector Machine (SVM), and Convolutional Neural Network (CNN). These models were selected to provide a comprehensive comparison between conventional statistical classifiers and deep learning‐based feature learning approaches. LDA and SVM represent widely used chemometric modeling strategies for hyperspectral classification, whereas CNN was employed to automatically extract nonlinear spectral features and investigate its potential for complex frost‐damage pattern recognition.

Samples were divided once into a calibration set and an independent prediction set at an approximate 7:3 ratio, stratified by the five temperature‐treatment classes. The calibration set contained 300 kernels (60 per class), and the prediction set contained 105 kernels (21 per class). The prediction dataset was completely excluded during model optimization to ensure an unbiased evaluation of classification capability. The optimal classification strategy was determined by comparing the prediction performance of different preprocessing methods, characteristic wavelength selection approaches, and classification algorithms. Because each cultivar contributed one kernel to every treatment class, cultivar identity was not used as a grouping variable, and kernels from the same cultivar could occur in both sets. The reported metrics therefore evaluate held‐out kernels under the present acquisition batch, not cultivar‐independent or production‐batch‐independent generalization.

#### 2.4.1 LDA

The core principle of LDA is to map high‐dimensional input features to a low‐dimensional space through linear transformation, so that samples of different classes are separated as much as possible and samples of the same class are clustered as much as possible, and then classification is realized based on low‐dimensional features (Li and Cai 2024). Its core idea can be summarized as “maximizing inter‐class distance and minimizing intra‐class distance.” Specifically, LDA first calculates the sample mean vector of each class to reflect the center position of samples of each class; then calculates the intra‐class scatter matrix (measuring the dispersion degree of samples of the same class) and the inter‐class scatter matrix (measuring the distance between the centers of samples of different classes) (Guo et al. 2023). These two matrices are defined as follows:

(3) $$S_{b}=\sum_{k=1}^{c}n_{k}\left(\mu_{k}-\bar{\mu }\right){\left(\mu_{k}-\bar{\mu }\right)}^{T}$$

(4) $$S_{w}=\sum_{k=1}^{c}\sum_{i=1}^{n_{k}}\left(x_{k}^{i}-\mu_{k}\right){\left(x_{k}^{i}-\mu_{k}\right)}^{T\;}$$

where $n_{k}$ is the number of samples in the k‐th class, $x_{k}^{i}$ is the *i*‐th sample of the *k*‐ th class, $\mu_{k}$ is the mean vector of the *k*‐th class, and $\bar{\mu }$ is the mean vector of all samples. Finally, by solving the eigenvalues and eigenvectors of the scatter matrix, the optimal linear transformation direction (the direction corresponding to the eigenvector) is found. The objective function to maximize the ratio of inter‐class scatter to intra‐class scatter is given by:

(5) $$\max_{P}\;\mathrm{tr}\left(\frac{P^{T}S_{b}P}{P^{T}S_{w}P}\right)$$

where P is the linear transformation matrix and $\mathrm{tr}\left(\cdot \right)$ denotes the trace operator. This direction can maximize the ratio of interclass scatter to intra‐class scatter after transformation, thus achieving the optimal class separation effect. After the mapping is completed, new samples can be classified by calculating their projection in the low‐dimensional space and the distance from the center of each class.

#### 2.4.2 SVM

The core principle of SVM is to find an optimal decision boundary (hyperplane) in the feature space, so that samples of different classes are clearly separated, and the distance (margin) from the points closest to the hyperplane (support vectors) in the two classes of samples to the hyperplane is maximized, that is, “maximum margin classification” (Huang et al. 2018). The classic binary linear SVM classifier can be expressed as the following function:

(6) $$f\left(x_{i}\right)=y_{i}=\mathrm{sgn}\left(\sum_{i=1}^{l_{n}}y_{i}\alpha_{i}\left(x_{i}^{T}\cdot x\right)+b\right)$$

where denotes the feature vector of the test sample, is the feature vector of the *i*‐th training sample, represents the class label of the training sample, is the number of support vectors, stands for Lagrange multiplier, *b* is the bias term of the hyperplane, and refers to the sign function for category judgment. For linearly separable samples, SVM finds the hyperplane that satisfies the classification conditions and has the maximum margin by solving a convex quadratic programming problem. This hyperplane is uniquely determined by the support vectors and has nothing to do with other nonsupport vector samples. For linearly inseparable samples, SVM maps low‐dimensional linearly inseparable features to a high‐dimensional feature space by introducing kernel functions (such as linear kernel, polynomial kernel, Gaussian kernel, etc.), so that the samples become linearly separable in the high‐dimensional space, and then constructs the maximum margin hyperplane based on the high‐dimensional space (Maggioni and Spinelli 2025). The kernel function used for feature mapping is defined as follows:

(7) $$\;K\left(x_{i},x_{j}\right)=\phi \left(x_{i}\right)\cdot \phi \left(x_{j}\right)$$

where $K\left(x_{i},x_{j}\right)$ is the kernel function, and $\phi \left(\cdot \right)$ indicates the nonlinear transformation function that maps original features into a high‐dimensional space. At the same time, in order to deal with noise samples, SVM introduces slack variables, allowing a small number of samples to be misclassified, so as to balance the margin size and classification error and improve the generalization ability of the model.

#### 2.4.3 CNN

**FIGURE 4:**
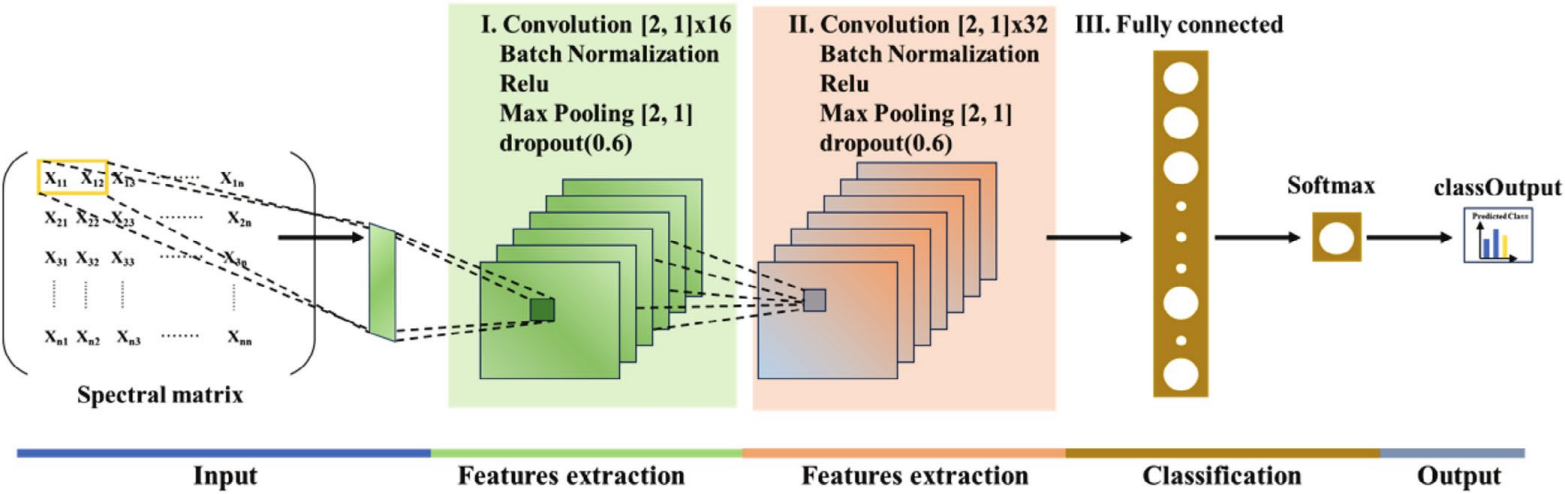
Structural diagram of the CNN model.

A one‐dimensional convolutional neural network (1D‐CNN) was developed in this study to automatically extract sequential spectral features and classify maize kernels from the five temperature‐treatment classes (Figure 4). Unlike traditional machine learning methods that require manually selected spectral descriptors, CNN can directly learn local spectral response patterns associated with frost‐induced physicochemical changes through hierarchical feature extraction.

The network adopted a cascaded convolution‐pooling architecture. Two convolutional layers with 2 × 1 convolution kernels were employed to extract local spectral features layer by layer, generating 16 and 32 feature channels, respectively. Batch normalization and ReLU activation functions were applied after each convolutional layer to accelerate convergence and improve nonlinear feature representation.

Subsequently, max‐pooling layers with a stride of 1 were introduced for feature compression and important information retention. The shallow convolutional layers mainly captured local variations in spectral peaks and valleys, whereas deeper layers gradually integrated these local patterns into higher‐level representations associated with different frost damage categories.

To reduce overfitting caused by the limited number of experimental samples, dropout regularization with a rate of 0.6 was applied in the fully connected layers. Finally, extracted features were transferred to a fully connected layer containing five neurons, and the Softmax function was used to calculate classification probabilities corresponding to the five temperature treatment groups.

The model was trained using the Adam optimizer with an initial learning rate of 1 × 10^−3^, a mini‐batch size of 64, and a maximum of 500 epochs. L2 regularization with a coefficient of 1 × 10^−4^ was applied. The learning rate was reduced by a factor of 0.5 every 450 epochs.

CNN training used a single fixed data split and one training run under the stated configuration. Random initialization can affect the fitted parameters, but repeated training runs, repeated random splits, and confidence intervals were not obtained. The reported values are therefore point estimates for this split rather than estimates of run‐to‐run variability. All model comparisons used the same calibration and prediction samples and the same stated training settings.

#### 2.4.4 Model Evaluation

Model performance was evaluated using multiple classification indicators, including accuracy, precision, recall, F1‐score, and confusion matrices. These evaluation metrics were selected because frost‐damage identification involves multi‐class classification, where both overall accuracy and class‐specific recognition capability are important. Accuracy represents the proportion of correctly classified samples among all samples, whereas precision reflects the reliability of positive predictions. Recall evaluates the ability of the model to identify samples belonging to each category, and F1‐score provides a balanced measurement between precision and recall. The confusion matrix was further used to visualize classification performance among different frost treatment groups and identify potential misclassification patterns. A comprehensive evaluation framework combining quantitative metrics and confusion matrix analysis was adopted to ensure a reliable assessment of model discrimination ability. All hyperspectral image processing, spectral analysis, and model construction procedures were performed using MATLAB R2023a (MathWorks Inc., Natick, Massachusetts, USA).

## 3 Results and Discussion

### 3.1 Spectral Characteristics

**FIGURE 5:**
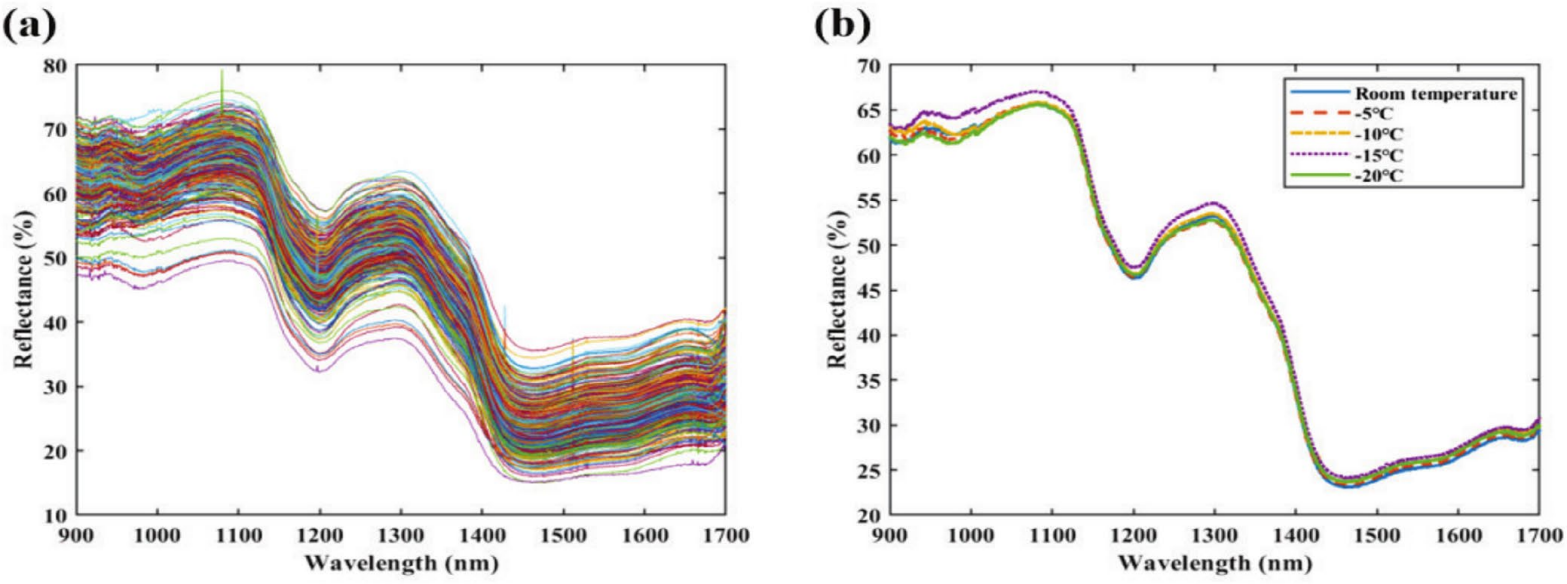
Raw (a) and average (b) reflectance spectra of maize samples under different temperatures.

To investigate spectral differences in maize samples at different temperatures, this study analyzed the average reflectance spectra of samples across five temperature gradients. Figure 5a shows the raw spectra in the 900–1700 nm wavelength range, while Figure 5b presents the average reflectance spectral characteristics of the samples at different temperatures.

The raw spectra in Figure 5a exhibit similar overall trends. Noise is observed at the beginning (approximately 900 nm) and end (approximately 1700 nm) of the spectral curves, which is likely attributable to the spectral acquisition equipment and environmental factors. The reflectance is relatively high in the 900–1200 nm wavelength range, while it is lower and more variable in the 1200–1700 nm wavelength range. This variation indicates that the longer wavelength region contains more abundant molecular vibration information and is therefore more sensitive to physicochemical alterations induced by freezing stress.

As shown in Figure 5b, the average reflectance spectra of the samples at different temperatures exhibit consistent absorption peak positions. The −15°C group has the highest overall reflectance, followed by the −10°C, −5°C, and room temperature groups, while the −20°C group has the lowest reflectance. The non‐monotonic variation of spectral intensity among different temperature treatments suggests that frost injury is not simply proportional to temperature reduction, but is closely associated with the extent of cellular disruption, water redistribution, and molecular structural changes caused by freezing stress. Excessive freezing conditions may lead to severe cell collapse and increased internal structural heterogeneity, thereby altering the interaction between incident light and kernel tissues.

Absorption valleys near 1200 and 1450 nm, as well as the absorption peak near 1300 nm, are clearly distinguishable across all temperature groups. These peaks are associated with the vibrations of chemical bonds such as O–H, N–H, and C–H in the water, carbohydrates, and proteins of the maize samples (Zhang et al. 2025; Li et al. 2021). Specifically, the absorption characteristics around 1200 nm are mainly related to C–H stretching vibrations associated with organic compounds, while the absorption feature near 1450 nm is closely related to O–H bond vibration of water molecules. Because freezing treatment directly affects cellular water distribution and macromolecular organization, these characteristic bands provide sensitive spectral responses to frost‐induced structural damage.

The observed spectral differences among temperature‐treated groups demonstrate that hyperspectral imaging can capture subtle variations associated with frost damage at the molecular level. Although the spectral profiles showed considerable overlap among different treatments, these hidden differences provide valuable information for subsequent feature extraction and classification modeling. These temperature‐induced spectral differences provide a basis for subsequent modeling to distinguish samples under different temperature conditions.

### 3.2 Performance of Prediction Models Based on the Full Spectrum

**FIGURE 6:**
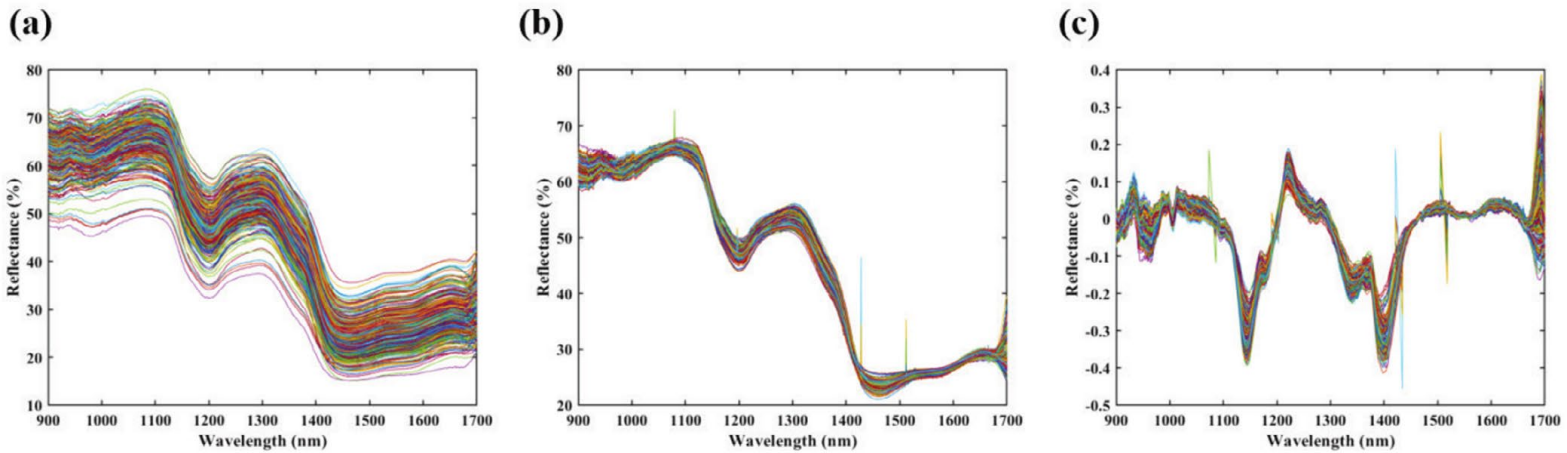
Hyperspectral reflectance spectra of pretreated maize samples: (a) SG smoothing; (b) MSC; (c) 1st derivative.

To eliminate interference from instrument noise, sample scattering, and baseline drift in the raw spectra, and to improve the stability and accuracy of subsequent modeling, three preprocessing methods—SG smoothing, MSC, and 1st derivative—were applied to the raw hyperspectral data of maize samples. The preprocessed spectra are presented in Figure 6. SG smoothing effectively removed random noise from the raw spectra while retaining key absorption features near 1100 and 1400 nm. MSC mitigated baseline shifts induced by variations in sample physical state and standardized the spectral baseline across different samples. The 1st derivative further suppressed baseline drift, resolved overlapping absorption peaks, and converted the gradual reflectance variations in the raw spectra into distinct positive and negative characteristic peaks, thereby highlighting subtle spectral differences among samples. Among these preprocessing strategies, MSC is particularly suitable for maize kernel analysis because the physical morphology, surface roughness, and internal structure of kernels may introduce significant scattering effects during hyperspectral acquisition. By correcting multiplicative and additive variations caused by sample heterogeneity, MSC enhances the comparability of spectral responses and improves the contribution of chemically meaningful information to subsequent classification models. Collectively, these preprocessing approaches improved spectral data quality from multiple perspectives, laying a reliable data foundation for the subsequent development of high‐precision classification models.

Table 1 summarizes the classification precision, recall, and accuracy of three models—LDA, SVM, and CNN on the calibration and independent prediction sets under four preprocessing strategies: Raw, SG smoothing, MSC, and 1st derivative. The results obtained from the calibration set and independent prediction set exhibited different trends, indicating that evaluation based solely on training performance is insufficient for determining the practical applicability of hyperspectral classification models.

**TABLE 1:** Classification results of LDA, SVM and CNN models based on different pretreatment methods.

Preprocessing methods	Model	Calibration set			Prediction set		
		Precision	Recall	Accuracy	Precision	Recall	Accuracy
Raw	LDA	1.000	1.000	1.000	0.9300	0.9332	0.9324
	SVM	0.6629	0.6527	0.6590	0.3876	0.4033	0.3900
	CNN	1.000	1.000	1.000	0.8877	0.8948	0.8900
SG	LDA	1.000	1.000	1.000	0.9400	0.9409	0.9424
	SVM	0.6629	0.6527	0.6590	0.3876	0.4033	0.3900
	CNN	1.000	1.000	1.000	0.8813	0.8854	0.8800
MSC	LDA	1.000	1.000	1.000	0.9600	0.9616	0.9589
	SVM	1.000	1.000	1.000	0.8867	0.8779	0.8800
	CNN	1.000	1.000	1.000	0.9379	0.9379	0.9400
1st derivative	LDA	1.000	1.000	1.000	0.9200	0.9169	0.9234
	SVM	0.9840	0.9838	0.9836	0.9111	0.9154	0.9100
	CNN	1.000	1.000	1.000	0.9239	0.9117	0.9200

All models achieved precision, recall, and accuracy values close to 1.000 on the calibration set under most preprocessing methods, which indicates that the models were capable of effectively learning the spectral patterns associated with different temperature treatments. However, the higher prediction errors observed in some models suggest that the complexity of hyperspectral data requires careful optimization to improve model generalization rather than simply increasing fitting capability.

In contrast, the results on the prediction set directly reflect the model's generalization ability, and significant differences exist among the different preprocessing methods. Raw spectra and SG‐smoothed spectra resulted in extremely poor generalization for the SVM model, with a prediction accuracy of only 0.3900, while LDA and CNN achieved moderate prediction performance. This phenomenon may be attributed to the fact that raw hyperspectral signals contain not only frost‐related chemical information but also irrelevant variations caused by sample scattering, illumination fluctuation, and instrumental noise. These disturbances can weaken the classification boundary, particularly for models that rely heavily on feature‐space separation.

After 1st derivative preprocessing, the generalization ability of all models improved to some extent: the SVM's prediction accuracy increased to 0.9100, while LDA and CNN reached 0.9234 and 0.9200, respectively. The improvement demonstrates that derivative transformation effectively enhances local spectral variation information by removing baseline shifts and emphasizing subtle changes in absorption behavior. This is particularly beneficial for identifying weak spectral responses caused by freezing‐induced molecular rearrangement.

Notably, MSC preprocessing achieved the best overall performance across all three models. The LDA model achieved the highest prediction accuracy of 0.9589, with precision and recall of 0.9600 and 0.9616, respectively, outperforming all other model‐preprocessing combinations. The SVM model, which performed the worst on both raw and SG spectra, saw its prediction accuracy significantly improve to 0.8800 after MSC. The CNN model also achieved an excellent prediction accuracy of 0.9400, outperforming its performance under raw, SG, and first‐derivative preprocessing.

The superior performance of MSC can be explained by its ability to reduce spectral variability caused by physical differences among maize kernels. Frost damage may alter kernel morphology, surface structure, and internal water distribution, which introduces additional scattering effects during spectral acquisition. By correcting these nonchemical variations, MSC enhances the relationship between spectral features and frost‐induced biochemical changes, thereby improving the reliability of classification models.

Although LDA achieved slightly higher accuracy than CNN when using full‐spectrum data after MSC preprocessing, the CNN model showed stronger potential for subsequent feature‐level optimization because of its ability to automatically extract nonlinear relationships from high‐dimensional spectral information. This characteristic becomes particularly important when combined with advanced feature extraction methods such as 2D‐COS, where complex spectral interactions need to be effectively represented.

The above results indicate that MSC preprocessing effectively eliminates interference from sample scattering and baseline drift, maximizes the discriminative power of features related to sample composition, and significantly enhances the generalization ability of classification models. Therefore, MSC was selected as the optimal preprocessing strategy for subsequent characteristic wavelength extraction and deep learning modeling in this study.

### 3.3 Performance of Prediction Models Based on Different Wavelength Section Algorithms

**FIGURE 7:**
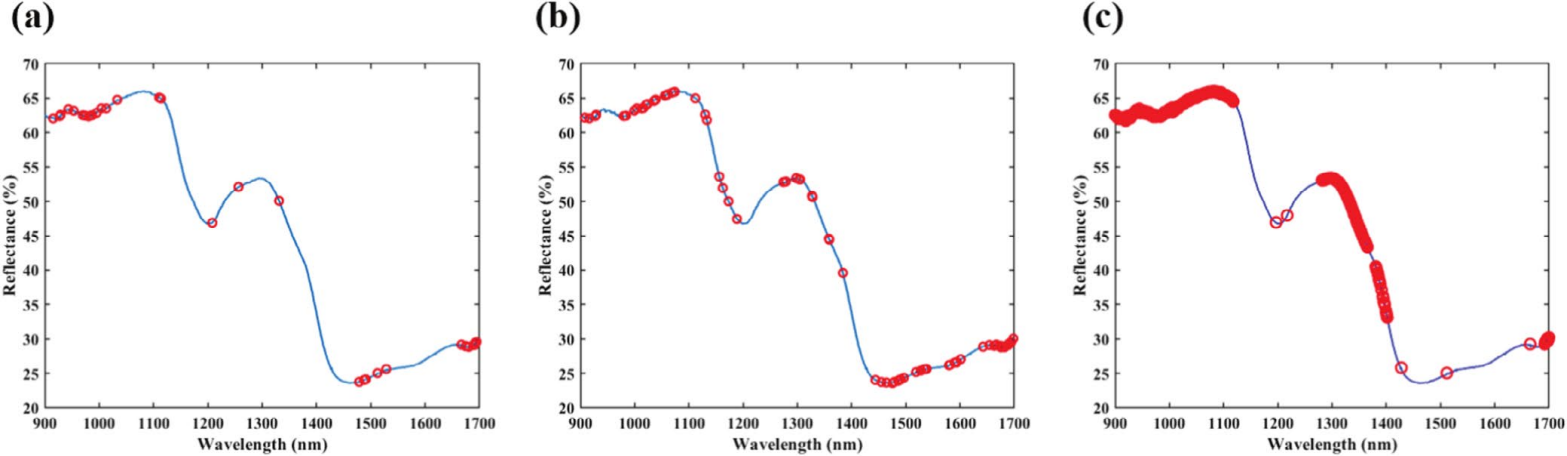
Distribution of selected characteristic wavelengths by different algorithms: (a) CARS; (b) VCPA‐IRIV; (c) 2D‐COS.

As shown in Figure 7, the three feature wavelength selection algorithms produced significantly different variable distributions for the maize hyperspectral data. These differences reflect the distinct feature mining mechanisms of each algorithm. CARS and VCPA‐IRIV mainly focus on eliminating redundant variables based on statistical importance, whereas 2D‐COS identifies characteristic wavelengths according to their dynamic response to external temperature perturbation, providing additional information related to molecular structural changes induced by frost stress.

The CARS algorithm identified a total of 39 feature wavelengths, with its effective variables primarily concentrated in the high‐reflectance regions of 900–1100 and 1300–1700 nm. It specifically retained the core absorption features related to maize chemical composition within the 1100–1300 nm range, achieving substantial dimensionality reduction while precisely identifying the key spectral intervals associated with components such as starch and protein. The relatively small number of selected wavelengths indicates that CARS effectively removed redundant information; however, excessive compression may also lead to the loss of weak but meaningful spectral responses associated with gradual biochemical changes during frost damage.

The VCPA‐IRIV algorithm identified 74 characteristic wavelengths. The selected variables covered the main reflectance region of 900–1200 nm and the absorption valley region of 1300–1500 nm. The distribution of characteristic points was denser and uniform, with significantly improved accuracy in identifying the carbohydrate absorption peak near 1150 nm, achieving a good balance between the number of features and information completeness. Compared with CARS, VCPA‐IRIV retained more informative variables, which improved the representation of spectral differences among temperature‐treated samples. However, this method still relies primarily on variable importance evaluation in the original spectral domain and may not fully capture the asynchronous response characteristics of overlapping absorption bands.

The 2D‐COS algorithm extracted a total of 432 characteristic wavelengths. This algorithm significantly highlighted the strong absorption regions within the 1100–1400 nm range. The selected wavelengths are densely distributed at characteristic absorption peaks and absorption valleys, comprehensively preserving the rich details in the maize sample spectra and effectively focusing on key spectral segments highly correlated with moisture and carbohydrate content.

Unlike conventional wavelength selection methods that independently evaluate the contribution of individual variables, 2D‐COS considers the correlation and sequential response of spectral signals under temperature perturbation. The synchronous spectrum reflects the overall sensitivity of spectral variables to freezing stress, while the asynchronous spectrum provides additional information regarding the different response orders of molecular groups. Therefore, the larger number of selected wavelengths does not simply represent redundant information, but rather preserves a multidimensional spectral response pattern associated with frost‐induced physicochemical changes.

The enrichment of characteristic variables in the 1100–1400 nm region is consistent with the spectral characteristics of maize kernels, where O–H, C–H, and N–H related vibrations contribute to signals associated with water migration, carbohydrate structure, and protein variation. These molecular changes are directly related to freezing injury, including membrane disruption, starch structural alteration, and protein denaturation. Therefore, 2D‐COS provides a more comprehensive representation of frost damage‐related spectral information compared with traditional wavelength selection approaches.

Table 2 summarizes the classification performance of LDA, SVM, and CNN models based on three characteristic wavelength selection methods (CARS, VCPA‐IRIV, 2D‐COS), evaluated by precision, recall, and accuracy on both calibration and prediction sets.

**TABLE 2:** Performance of quantitative models based on different characteristic wavelength selection algorithms.

Preprocessing methods	Model	Calibration set			Prediction set		
		Precision	Recall	Accuracy	Precision	Recall	Accuracy
CARS	LDA	0.9435	0.9410	0.9410	0.9810	0.9800	0.9800
	SVM	0.8954	0.8951	0.8951	0.9375	0.9299	0.9300
	CNN	0.9753	0.9735	0.9738	0.9609	0.9599	0.9600
VCPA‐IRIV	LDA	0.9835	0.9835	0.9836	0.9624	0.9587	0.9600
	SVM	0.9381	0.9374	0.9377	0.9101	0.9090	0.9100
	CNN	0.9934	0.9933	0.9934	0.9293	0.9306	0.9300
2D‐COS	LDA	1.000	1.000	1.000	0.9126	0.8100	0.7990
	SVM	0.9777	0.9772	0.9770	0.9576	0.9618	0.9600
	CNN	0.9969	0.9969	0.9967	0.9742	0.9647	0.9700

For the CARS‐based models, the CARS‐LDA combination achieved the highest prediction accuracy of 0.9800 among all groups. This result indicates that compact spectral features selected by CARS contain sufficient discriminative information for linear classification under certain conditions. However, the performance advantage of CARS was mainly observed in combination with LDA, suggesting that the selected variables may mainly represent relatively independent spectral differences rather than complex nonlinear relationships.

The VCPA‐IRIV‐based models showed moderate overall performance, with the VCPA‐IRIV‐LDA model reaching a prediction accuracy of 0.9600, while the SVM and CNN models achieved 0.9100 and 0.9300, respectively. The relatively stable performance of VCPA‐IRIV demonstrates its ability to preserve informative variables while reducing spectral redundancy, but its limited improvement compared with 2D‐COS suggests that static variable screening may not fully describe the dynamic spectral response induced by freezing stress.

Notably, the 2D‐COS‐CNN model delivered the optimal comprehensive prediction performance: it attained a prediction accuracy of 0.9700, with precision and recall of 0.9742 and 0.9647, outperforming all other model‐feature selection combinations. Meanwhile, the 2D‐COS‐CNN model maintained a high calibration set accuracy of 0.9967, showing strong fitting and held‐out prediction performance for this single split.

The superior performance of the 2D‐COS‐CNN model can be attributed to the complementary advantages of both components. 2D‐COS enhances the sensitivity of spectral features by transforming subtle temperature‐induced variations into highly discriminative correlation patterns, while CNN further extracts nonlinear relationships among these high‐dimensional spectral variables through hierarchical feature learning. This combination enables effective characterization of complex relationships between spectral responses and the imposed temperature‐treatment classes.

Compared with traditional machine learning models, which generally depend on manually selected features and predefined decision boundaries, CNN can automatically capture local spectral patterns, including peak shifts, intensity variations, and wavelength interactions. Therefore, the integration of 2D‐COS and CNN provides a more powerful strategy for identifying frost‐related quality deterioration in maize kernels.

For the present single‐split dataset, 2D‐COS‐CNN provided the highest combined CNN precision, recall, and accuracy among the evaluated feature‐selection pipelines. This result supports its promise for the current samples but does not by itself establish cultivar‐independent, batch‐independent, or deployment‐level generalization.

## 4 Discussion

**FIGURE 8:**
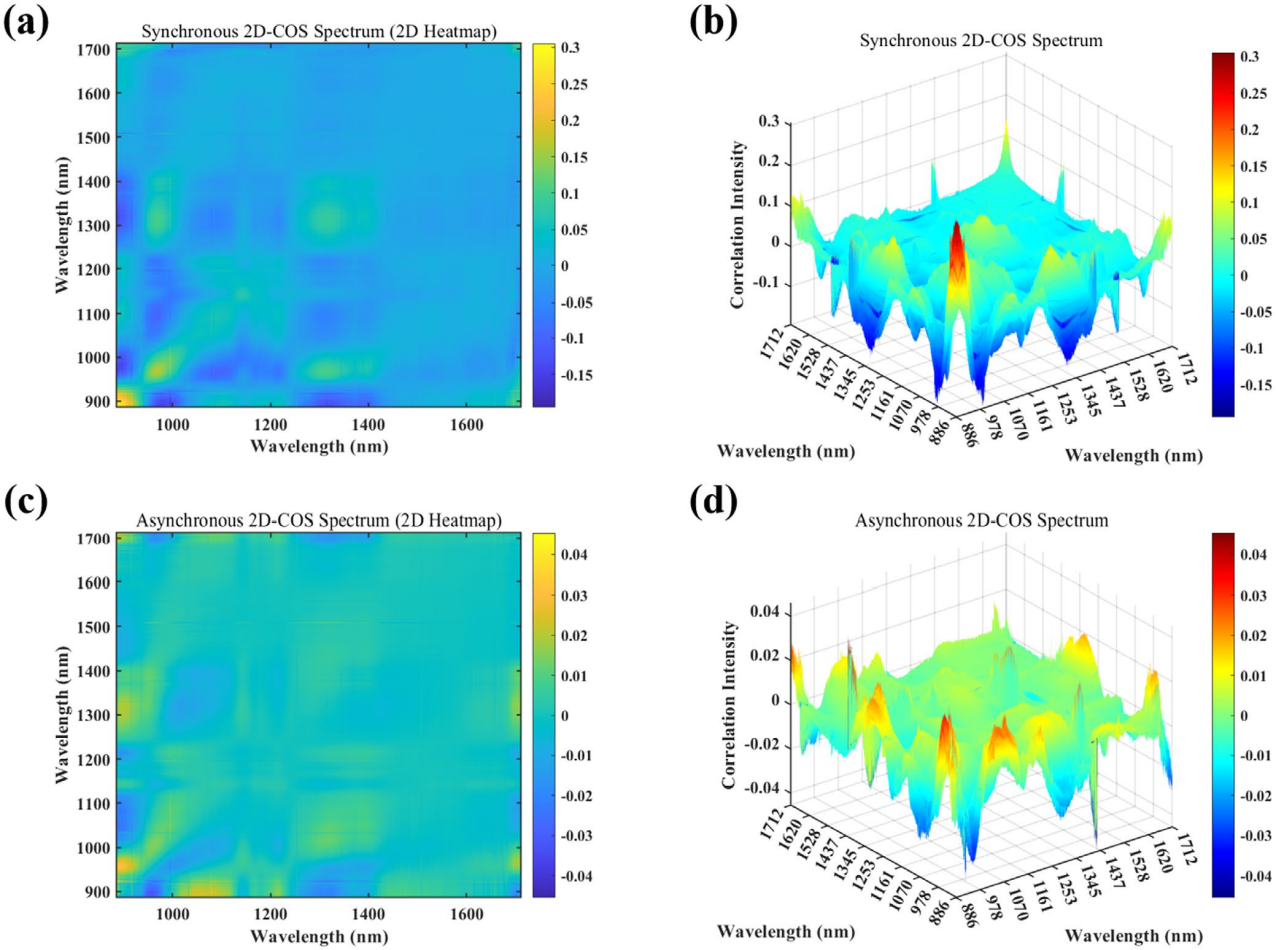
Two‐dimensional correlation spectroscopy (2D‐COS) maps of maize samples. (a) 2D heat map of synchronous spectrum. (b) 3D surface plot of synchronous spectrum. (c) 2D heat map of asynchronous spectrum. (d) 3D surface plot of asynchronous spectrum.

The outstanding performance of the 2D‐COS‐CNN model originates primarily from the inherent mechanistic advantages of two‐dimensional correlation spectroscopy (2D‐COS) in mining complex physicochemical characteristics. Within the near‐infrared range of 900–1700 nm, frost‐induced biochemical alterations in maize kernels, including water migration caused by cell membrane damage and the degradation of starch and proteins, are generally reflected in subtle vibrational variations of O–H and C–H bonds. Such weak signals present severe spectral peak overlap in conventional one‐dimensional spectra. Therefore, direct interpretation of conventional spectra may fail to fully capture the physicochemical responses associated with the ordered temperature treatments. By introducing temperature perturbation, the 2D‐COS algorithm maps one‐dimensional spectra into a two‐dimensional space as shown in Figure 8. The synchronous plot identifies frost‐sensitive autocorrelation peaks, while the asynchronous plot resolves phase differences among overlapping spectral peaks. This transformation enhances spectral resolution by revealing the sequential response behavior of different chemical components under thermal stress, thereby providing additional mechanistic information beyond conventional spectral analysis.

The temperature classes are experimental exposure labels, not direct measurements of damage severity. The spectral regions discussed above are consistent with water‐, carbohydrate‐, protein‐, and lipid‐related absorptions, but this study did not measure moisture redistribution, starch pasting, or gelatinization, protein functionality, lipid oxidation, or membrane integrity in the same kernels. Consequently, these mechanistic links should be treated as hypotheses supported by prior literature, and the present data demonstrate optical discrimination of treatment classes rather than quantitative prediction of a specific physicochemical damage index.

As illustrated in Table 2, the 432 characteristic wavelengths screened by 2D‐COS exhibit abundant information richness in the strong absorption region of 1100–1400 nm, which substantially outperforms the feature representation capacity of CARS (39 variables) and VCPA‐IRIV (74 variables). Although a larger number of variables were retained, these features were not simply redundant spectral points, but represented wavelength regions with strong responses to temperature‐induced molecular changes. The retained bands were mainly distributed around regions associated with water, carbohydrate, and protein‐related absorptions, which are highly relevant to frost‐induced structural and compositional alterations in maize kernels. This approach not only eliminates interference from background noise but also physically amplifies subtle spectral differences among samples with varying frost damage severities, thereby providing high‐signal‐to‐noise ratio and highly discriminative feature inputs for subsequent deep learning modeling. Compared with traditional variable selection strategies that mainly rely on statistical correlations, 2D‐COS provides a perturbation‐driven feature mining strategy, which is more suitable for identifying weak but biologically meaningful spectral responses caused by external stress.

The relatively large 432‐wavelength subset also requires caution. The normalized‐score threshold of 0.5 was a fixed operational rule, but its sensitivity and the recurrence of individual wavelengths across resampled calibration sets were not tested. In addition, detector‐edge noise near 900 and 1700 nm was observed but not removed by a predefined exclusion band. These issues limit claims that every selected wavelength is stable or mechanistically specific, even though the complete subset yielded strong prediction performance in the current split.

Furthermore, the convolutional neural network (CNN) can learn nonlinear local patterns that differ from those represented by the evaluated LDA and SVM models when processing high‐dimensional feature data with local correlation. As summarized in Table 1, under MSC preprocessing, the test accuracy of the CNN increased from 0.8900 using raw spectra to 0.9400. When further combined with 2D‐COS feature optimization (Figure 8), the prediction set accuracy was further improved to 0.9700, with a recall rate reaching 0.9647. These improvements indicate that spectral preprocessing and feature enhancement play complementary roles: MSC reduces physical scattering variations caused by sample heterogeneity, whereas 2D‐COS extracts stress‐responsive spectral information associated with frost damage progression.

The unique local receptive field and hierarchical feature abstraction mechanism constitute the core strengths of CNN. Convolutional layers automatically extract local morphological traits of spectral curves, such as the slope and curvature of absorption peaks, whereas pooling layers strengthen the model's robustness against spectral drift. In contrast, the support vector machine (SVM) yielded an accuracy of merely 0.3900 with raw and SG‐preprocessed spectra, indicating severe underfitting. This difference suggests that frost damage discrimination involves complex nonlinear relationships between spectral variations and physiological changes, which cannot be fully characterized by conventional linear or shallow machine learning models. However, the available computer‐vision literature cannot substitute for validation on maize kernels: algorithms developed for generic edge extraction or small‐object detection address related image‐processing problems but do not establish performance under kernel‐specific illumination, morphology, or spectral variation (Al‐Ghaili et al. 2020; He et al. 2023).

The 432 feature points derived from 2D‐COS construct a sufficient information space to support in‐depth feature learning of CNN. Through multi‐layer nonlinear transformation, the model effectively characterizes the intricate mapping relationships between spectral reflectance fluctuations and frost damage grades. The integration of perturbation‐enhanced spectral representation and deep feature extraction enables the model to achieve a balance between feature richness and classification robustness. The strategy integrating high‐dimensional refined features and deep network discrimination effectively balances model learning capability and generalization performance, providing a solid technical foundation for rapid, nondestructive food quality screening of frost‐damaged maize kernels. From an application perspective, this approach provides a potential solution for intelligent quality monitoring of frost‐affected grains during postharvest handling, storage, and processing. The observed accuracy should therefore be regarded as internal evidence from one controlled acquisition batch. Robust deployment will require naturally frost‐damaged kernels, cultivar‐grouped evaluation, repeated training and resampling, independent production batches, and tests under online sorting conditions. Transfer‐learning strategies may help address batch and domain shifts, but their benefit must be demonstrated directly on the target grain‐imaging system (Su et al. 2025).

## 5 Conclusion

This study established a nondestructive detection framework for food quality assessment of frost‐damaged maize kernels by integrating hyperspectral imaging (900–1700 nm) with deep learning. MSC effectively reduced spectral variability and improved model stability, while 2D‐COS extracted 432 frost‐responsive characteristic bands by resolving overlapping spectral signals. By incorporating temperature perturbation into spectral analysis, 2D‐COS enhanced the detection of weak molecular changes associated with freezing stress. The proposed 2D‐COS‐CNN model achieved a prediction accuracy of 0.9700, a recall of 0.9647, and a calibration accuracy of 0.9967, demonstrating strong discrimination among the five controlled temperature‐treatment classes in the present single‐split dataset. The combination of perturbation‐enhanced feature mining and deep nonlinear learning provides a promising strategy for rapid quality screening of frost‐affected maize kernels in food processing and supply chains. Future studies should further establish quantitative relationships between spectral features and specific quality indicators, such as starch pasting properties and protein functionality, to improve the practical application of hyperspectral technology in food quality evaluation. These findings do not yet establish natural frost‐damage grading or external generalization because the labels were defined by treatment temperature, reference physicochemical damage measurements were unavailable, cultivar identity was not isolated between sets, and repeated runs were not performed. Future work should validate naturally frost‐damaged kernels from independent production batches, quantify relevant moisture, starch, protein, lipid, and membrane indicators, evaluate feature‐selection stability, and test real‐time performance under online sorting conditions.

## Author Contributions

**Dingguo Wang:** investigation, validation, resources, writing – original draft. **Jiaxuan Nan:** conceptualization, methodology, software, investigation, validation, formal analysis, data curation, writing – original draft. **Jiaxuan Li:** software, visualization, writing – review and editing. **Wuping Zhang:** conceptualization, methodology, supervision, project administration, funding acquisition. **Huihui Wang:** investigation, validation, formal analysis. **Jiwan Han:** conceptualization, methodology, supervision, project administration, funding acquisition. **Fuzhong Li:** conceptualization, methodology, supervision, project administration, funding acquisition. **Changxu Hu:** validation, visualization, writing – review and editing.

## Funding

This work was supported by the National Administration of Foreign Experts Affairs International Cooperation Project (grant no. G2022004004L) and the Key Research and Development Program of Shanxi Province (grant no. 202202140601021).

## Conflicts of Interest

The authors declare no conflicts of interest.

## Data Availability

The data that support the findings of this study are available from the corresponding author upon reasonable request.
